# The Composition, Physicochemical Properties, Antioxidant Activity, and Sensory Properties of Estonian Honeys

**DOI:** 10.3390/foods10030511

**Published:** 2021-03-01

**Authors:** Evelin Kivima, Kristel Tanilas, Kaie Martverk, Sirli Rosenvald, Loreida Timberg, Katrin Laos

**Affiliations:** 1 Department of Chemistry and Biotechnology, Tallinn University of Technology, Akadeemia tee 15, 12618 Tallinn, Estonia; kaie.martverk@gmail.com (K.M.); katrin.laos@ttu.ee (K.L.); 2 Center of Food and Fermentation Technologies (CFFT), Akadeemia tee 15a, 12618 Tallinn, Estonia; kristel@tftak.eu (K.T.); Sirli@tftak.eu (S.R.); 3 Estonian Maritime Academy, Tallinn University of Technology, Kopli 101, 11712 Tallinn, Estonia; loreida.timberg@taltech.ee

**Keywords:** honey, polyphenols, flavonoids, antioxidant activity, amino acids, sensory analysis, flavor, aroma, CIELAB

## Abstract

Thirty honey samples from different regions of Estonia were investigated to determine the chemical compositions, physicochemical properties, bioactive compounds, and sensory characteristics of typical honeys from a northern climate. The physicochemical parameters, such as electrical conductivity, moisture content, free acidity, hydroxymethylfurfural, diastase, and invertase activity were measured. The color was measured and expressed by L*-, a*-, and b*-coordinates. Sensory parameters were determined by using “fruity”, “floral”, “berry-like”, “herbal”, “woody”, “spicy”, “sweet”, and “animal-like” as the main odor and flavor attributes. The total polyphenol and flavonoid contents were in the range of 26.2–88.7 mg gallic acid equivalents (GAE) per 100 g and 1.9–6.4 mg quercetin equivalents (QE) per 100 g, respectively. The identified polyphenols showed the highest intensities of caffeic acid, coumaric acid, and abscisic acid and its derivatives. The protocatechuic acid intensity was highest in honeys containing traces of honeydew elements and of cinnamic acid and myricetin in heather honey. The water-soluble antioxidant values were 37.8–311.2 mg ascorbic acid equivalents (AAE) per 100 g and the lipid soluble antioxidant values were 14.4–60.7 mg Trolox equivalents (TE) per 100 g. The major amino acid in the analyzed honeys was proline, with variable values depending on the honey’s botanical source. Correlations were calculated based on the results obtained. It was revealed that the typical Estonian honey has floral, berry-like, sweet, and rather mild sensory characteristics. Most of the honeys lacked stronger spicy, woody, and animal-like attributes. The typical color of Estonian honey is quite light.

## 1. Introduction

Honey is a natural product containing about 600 different constituents [1]. It consists mainly of carbohydrates and water, and traces of other components, such as vitamins, minerals, and aromatic substances [2]. Honey is also rich in enzymatic (e.g., glucose oxidase and catalase) and non-enzymatic antioxidants, such as flavonoids (chrysin, pinocembrin, pinobanksin, quercetin, kaempferol, luteolin, galangin, apigenin, hesperetin, and myricetin), phenolic acids (caffeic, coumaric, ferulic, ellagic, and chlorogenic), organic acids, ascorbic acid, amino acids, proteins, Maillard reaction products, α-tocopherol and carotenoids [3,4,5,6,7]. Darker honeys have higher total polyphenol and flavonoid values [5,8], and the polyphenol level in honey is directly associated with flower nectar, propolis, and pollen [9]. High correlations between antioxidant activity and total polyphenol and flavonoid contents have been found in several studies [3,10,11]. Honey properties and compositions depend, above all, on the chemical content of the nectar of the plant that the honey is derived from, as well as on the geographic area, as soil and weather determine melliferous flora, bee species, and even storage mode [12].

From the consumer’s point of view, honey sensory properties, such as flavor, aroma, and color, are most important, and those parameters are determined by the honey’s botanical origin. In addition to chemical and pollen analysis, sensory analysis also provides an opportunity to evaluate the honey’s quality, making it possible to detect the presence of such defects as impurities, off-flavors and odors, which are indicators of changes happening during storage or heating during pasteurization [13,14]. Natural honey variability can make its sensory characterization complicated because in mixed botanical origin honeys the strong sensory characteristics of one botanical source, even in minor amounts, can affect the milder characteristics of another botanical source and change the overall sensory profile [15]. Nevertheless an analysis can reveal the presence of botanical components not picked up by other analytical systems (physicochemical or melissopalynological) [14].

Total honey production in Estonia is approximately 1100 tons per year [16]. Polyfloral honey is most common, as unifloral honey production in Estonia is challenging due to the short summers, small areas of certain plant types during the flowering period, and changing weather conditions. The most widespread plants in Estonia that provide both pollen and nectar are rapeseed (*Brassica napus*), white clover (*Trifolium repens*), melilot (*Melilotus officinalis*), raspberry (*Rubus idaeus*), and willow (*Salix* spp.), in addition to heather (*Calluna vulgaris*), which is one of the most highly valued honey plants [17]. 

There have been a few scientific studies on Estonian honeys investigating pesticide residues [18,19], pollen analysis [20], amino acid analysis [21], physicochemical properties [17,22], and crystallization behavior [23]; however, there has been no diverse and comprehensive survey on the quality, nutritional properties, and sensory characterization of typical Estonian honeys. Therefore, this work aims to determine the physicochemical properties, antioxidant activity, bioactive attributes, amino acid compositions, and sensory quality of honeys from different areas of Estonia. 

## 2. Materials and Methods

### 2.1. Honey Samples

Honey samples were provided directly by beekeepers from all Estonian counties and were stored for further analysis in a climate chamber (+18 °C) in the absence of light. All beekeepers were Estonian Beekeepers Association members. The honey samples were harvested from June to September. One honey (harvested at the beginning of October) was identified by the beekeeper as a unifloral heather honey. Honey sample botanical origins were confirmed by melissopalynological analysis.

### 2.2. Melissopalynological Analysis

Harmonized melissopalynology methods [24] were used in order to determine the honeys’ botanical origins. Honey (10 g) was dissolved in 20 mL of distilled water. The solution was centrifuged and the remaining liquid was removed. The sediment was used to make the microscope preparation. The relative frequency was found by counting at least 500 pollen grains. 

### 2.3. Physicochemical Parameters

The physicochemical parameters (electrical conductivity, moisture content, diastase activity, free acidity and invertase activity, and hydroxymethylfurfural) were determined using harmonized International Honey Commission methods [25]. The glucose and fructose levels were determined using an in-house developed HPLC-RI method. Briefly, the honey samples were diluted with water (25×), filtered, and injected into the HPLC system (Waters). A Zorbax Carbohydrate Analysis column (Agilent Technologies Inc., Santa Clara, CA, USA), a temperature of 30 °C, and isocratic elution with acetonitrile/water (75/25 *v/v*) were used to separate the sugars. The data were processed using Empower software (Waters, Milford, MA, USA).

The honey colors were measured by the CIELAB method using spectrophotometer CM-700d (Konica Minolta Inc., Osaka, Japan). The honey samples were heated to 50 °C, poured into petri dishes, covered with lids and left at room temperature for 30 min before the measurements. The measured honey was 1 cm thick. The L*-, a*-, b*-parameters were determined against a white background, readings were taken from three different points and the averages were calculated.

### 2.4. Amino Acids

Free amino acids were determined by the LC-UV methodology (AccQ•Tag™ Ultra Derivatization Kit; Waters, Milford, MA, USA) developed by Waters. Honey samples were dissolved in water, vortexed, and filtered (0.2 µm). The samples were derivatized with an AccQ-Fluor reagent (6-aminoquinolyl-N-hydroxysuccinimidyl carbamate) and then loaded on an AccQ-Tag Ultra column. Amino acids were separated using a gradient of AccQ-Tag Ultra eluents A and B. These were detected with a photodiode array detector, and data were processed with Empower 2 software (Waters, Milford, MA, USA).

### 2.5. Bioactive Compounds

#### 2.5.1. Total Polyphenol Content and Identification

The total phenolic content (TPC) of each sample was determined using the Folin-Ciocalteu method, according to Meda et al. [26]. Each honey sample (5 g) was diluted to 50 mL with distilled water and filtered through Whatman No. 1 paper. This solution (0.5 mL) was then mixed with 2.5 mL of 0.2 N Folin-Ciocalteu reagent (Sigma-Aldrich Chemie, Steinheim, Germany) for 5 min and 2 mL of 75 g L^−1^ sodium carbonate (Na_2_CO_3_) (Sigma-Aldrich Chemie, Steinheim, Germany) solution was then added. After incubation in the dark at room temperature for 2 h, the reaction mixture absorbance was measured at 760 nm against a methanol blank (Spectronic Helios Gamma UV-Vis Spectrophotometer, Thermo Fisher Scientific, Waltham, MA, USA). Gallic acid (Sigma-Aldrich Chemie, Steinheim, Germany) (0–200 mg L^−1^) was used as the standard to produce the calibration curve. The mean of three readings was used and the total phenolic content was expressed in mg of gallic acid equivalents (GAE) per 100 g of honey.

To identify the polyphenols in the honey, a liquid-chromatography-mass-spectrometry (LC-MS) method developed in the Center of Food and Fermentation Technologies was used. Polyphenols were isolated and pre-concentrated from honey samples using a solid-phase extraction (SPE) procedure, as described by Michalkiewicz et al. [27], with modifications. Briefly, honey samples were extracted with formic acid (pH < 2) and concentrated using an SPE column (Oasis HLB, Waters, Milford, MA, USA). The adsorbed compounds were eluted with methanol and dried using a SpeedVac evaporator at 30 °C. A methanol: water (1:1) mixture was used to reconstitute dried samples. Polyphenols were separated using an ACQUITY UPLC HSS C-18 1.8 μm (2.1 × 150 mm) column (Waters, Milford, MA, USA). Elution was carried out using water + 0.1% formic acid (*v/v*) (A) and a 0.1% acetonitrile (*v/v*) + 0.1% formic acid (*v/v*) (B) gradient: initial 86%A/14%B (*v/v*), 0–8 min 70%A/30%B (*v/v*), 8–18 min 55%A/45%B (*v/v*), 18–21 min 20%A/80%B (*v/v*), 21–22 min 100%B, 22–23 min 100%B, and 86%A/14%B (*v/v*) at a 0.25 mL min^−1^ flow rate. Mass spectrometry analysis was carried out in a negative electrospray ionization mode. Data were collected and reprocessed using MassLynx 4.1 software (Waters, Milford, MA, USA).

The detected polyphenols and their derivatives (D) (*m/z*) were numbered from 1 to 34 (Table 1). To evaluate the compounds’ indirect abundance in the honey samples, their mass spectra signal intensities were used.

#### 2.5.2. Total Flavonoid Content 

The total flavonoid content (TFC) was determined by the method described by Bueno-Costa et al. [28]. A honey solution (100 mg mL^−1^) was prepared with methanol 50% (*v/v*), previously homogenized, and filtered through a quantitative filter. Honey solution (5 mL) was mixed with 5 mL AlCl_3_ (2% *w/v*) in methanol. The mixture was homogenized and allowed to stand for 30 min in the dark. The absorbance was measured at 415 nm (Spectronic Helios Gamma UV-Vis Spectrophotometer, Thermo Fisher Scientific, Waltham, MA, USA). The total flavonoid content was determined using a standard curve with quercetin (Sigma-Aldrich Chemie, Steinheim, Germany) (0–50 mg L^−1^) as a standard. A three-reading mean was used and expressed as mg of quercetin equivalents (QE) per 100 g of honey.

#### 2.5.3. Antioxidant Activity

To evaluate the antioxidant activity, the photochemiluminescence (PLC) method, together with a Photochem device (Analytik Jena AG, Jena, Germany), was used. Commercial standard sets of total water-soluble antioxidant capacity (ACW) and total lipid soluble antioxidant capacity (ACL) and a method by Wesolowska and Dżugan [29] were used.

A honey solution (10 g L^−1^) dissolved in distilled water for ACW and in methanol for ACL was used; 20 µL of suitable solution was mixed with ready reagents (ACW or ACL) according to the attached instructions. The prepared mixture was placed in a Photochem device equipped with PCL Soft 5.1 software (Analytik Jena AG, Jena, Germany). The results were calculated on the basis of standard curves into mg ascorbic acid (AA) equivalents per 100 g of honey for ACW and mg Trolox equivalents (TE) per 100 g of honey for ACL.

### 2.6. Sensory Analysis

#### 2.6.1. Sample Preparation

Guidance for the sample preparation was taken from Piana et al. [14]. Honey preparation was done differently for gustatory and olfactory assessment. For flavor evaluation, about 30 g of honey was put in sampling containers (one for each assessor) and covered with twist-off caps. For odor evaluation, honey was diluted in a 1:1 portion by weight with odorless drinking water, and 20 mL of the honey-water mixture was put in sniffing glasses and covered with lids. The prepared samples were kept at room temperature for at least an hour before analyses to allow the headspace to equilibrate. All of the assessments were done between 10 a.m. and 12 p.m., and the room temperature was 21 ± 1 °C during the evaluations.

#### 2.6.2. Training of Assessors

Honey sample sensory evaluation was conducted under standardized conditions in a sensory room [30]. The panel consisted of 10 expert panelists between the ages of 25 and 40 from the Center of Food and Fermentation Technologies. All of the assessors had previous experience in sensory analysis, meeting the requirements described in ISO 8586:2012 [31]. The assessors participated in two training sessions to become familiar with the samples and took part in choosing identifying odors and flavors by using terminology from the odor and aroma wheel described by Piana et al. [14] and the Honey Flavor Wheel (UC Davis, Honey, and Pollination Center). During the discussion, the assessors were trained to use the given scales (0–15) and vocabulary based on EN ISO 13299:2016 [32].

#### 2.6.3. Sensory Evaluation

For both the honey flavor and odor evaluations, the following attributes were chosen to describe the samples: “berry-like”, “fruity”, “floral”, “herbal”, “woody”, “spicy”, “sweet”, and “animal-like”. Besides overall flavor and aroma intensities, sour taste levels were determined. A 0 to 15 scale was used for all assessments. The olfactory characteristics were evaluated first.

Water and crackers were used to cleanse the palate between sample evaluations. In each session, only six honey samples were analyzed to avoid fatigue. Sensory analyses were carried out in duplicate, for a total of 10 sessions. Average scores were calculated over two sessions and 10 panelists.

### 2.7. Statistical Analysis

For data analysis and visualization, Principal Component Analysis (PCA) was used and RStudio 1.0.136 (Boston, MA, USA) was applied. The data were normalized before carrying out the analysis. Pearson correlation coefficients were calculated from the measurements. Mean values were calculated for all sensory attributes over two sessions and 10 assessors. For statistical analysis, the R software packages FactoMineR and Factoextra were used (R 3.4.0.). Before the analysis, all data were auto-scaled.

## 3. Results and Discussion

### 3.1. Melissopalynological Analysis

All honeys, except for one, were determined to be polyfloral honeys and the pollen types were variable. The most dominant pollen types detected in honey samples were Cruciferae (mainly *Brassica napus*) and Rosaceae (mainly *Rubus* type). In addition, the pollens of willow (*Salix* spp.), clover (*Trifolium*), and alder buckthorn (*Frangula alnus*) occurred in noticeable amounts. By melissopalynological analysis, two honeys were observed to contain numerous honeydew element traces (numbers 28 and 29). The unifloral heather honey (number 17) identified by beekeepers was confirmed to be such by pollen analysis (*Calluna vulgaris*, 7%). 

### 3.2. Physicochemical Parameters 

Certain limits have been set on physicochemical quality parameters to avoid honey adulteration and to guarantee safe and good quality honey on the market [33]. The analyzed honeys’ physicochemical parameters are presented in Table 2. The moisture content varied from 15.6% to 20.9%, four honeys exceeding the 20% level set by Council Directive 2001/110/EC. This higher percentage may have resulted from processing techniques or storage conditions [2]. A higher heather honey moisture content (20.4%) is allowed [33].

The analyzed honeys’ measured electrical conductivities were all under 0.8 mS cm^−1^. The highest values (0.7 mS cm^−1^ and 0.8 mS cm^−1^, respectively) were observed in the heather honey (number 17) and honey number 23 (containing the highest alder buckthorn pollen level: 29%). The honeydew honeys’ electrical conductivity should be no less than 0.8 mS cm^−1^, and, in this case, the lower values of electrical conductivity of the honeys containing traces of honeydew elements (numbers 28 and 29) were probably due to their too small amounts. Free acidity is related to the decrease in honey quality as the level increases over 50 mmol kg^−1^ [33]. The investigated honeys’ free acidity levels were in the range of 12.0 to 43.0 mmol kg^−1^, which met the quality honey requirements. Invertase activity is not standardized in Estonia and can vary greatly, especially in summer honeys; however, the suggested level is at least 50 U kg^−1^ for fresh unheated honeys [34]. The invertase activity of the studied honey samples ranged from 50.4 U kg^−1^ to 231.0 U kg^−1^, which is within the fresh honey range.

The analyzed honeys’ diastase activity varied from 15.4 to 58.8 (Schade units) and the highest level was found in heather honey. However, all honeys met the quality norms. 

One of the most important quality indicators of honey is its hydroxymethylfurfural (HMF) level. All analyzed honeys proved to be of high quality, as the HMF concentrations were under 19.5 mg kg^−1^. 

The total fructose and glucose levels in all honey samples were above 60 g per 100 g and, thus, met the quality requirements. The fructose content was higher in all honey samples, with an average of 39.4 g per 100 g, than the glucose content, with an average of 34.8 g per 100 g. These results are similar to those of a survey conducted in our previous study [17]. In terms of fructose and glucose (F/G) ratio, honeys with levels of about 1.0 can be considered blossom honeys [35]. However, the heather honey F/G was 1.3, which is comparable to the heather honeys analyzed in our previous study [17] and by other authors [36,37]. Honey color is associated with phenolic compounds, pollen and mineral element contents [13], and depends directly on the plants the nectar is derived from [38]. The L*-coordinates, which indicate honey lightness or darkness, ranged from 65.3 to 90.4. The a*-coordinates (redness/greenness) and b*-coordinates (blueness/yellowness) were in the range of −1.7 to 12.5 and 25.6 to 60.3, respectively. Generally, most honeys were rather light in color and had red, yellow, and mildly green tones. The heather honey had the lowest L*-value and highest a*-value, which meant that it was the darkest and one of the most reddish honeys of the analyzed samples. The two honeys containing honeydew elements differed greatly from the other honeys. These two honeys were slightly lighter than the heather honey but tended to be the most reddish and yellowish, with the highest a*- and b*-values. Based on the calculated correlation coefficient between the L*- and a*-value (Table 3), it is clear that, as expected, the darker the honey, the more reddish tones it had.

### 3.3. Amino Acids

The UV chromatograms of amino acids of standard and honey sample (number 1) are shown in the Supplementary material (Figure S1). Honey quality, maturity, and natural origin are estimated by the proline content, which can also be considered an indicator of the total amounts of amino acids in honey [26]. In all of the honey samples, the proline concentration was higher than any other amino acid, followed by phenylalanine and glutamine (Table 4). The proline content ranged from 257 mg kg^−1^ to 1328 mg kg^−1^, which indicated good quality honeys, meeting the general requirement of the proline content being above 200 mg kg^−1^ [39].

According to Crane [40], the proline content is high in honeydew honeys. In this study, very high proline values were observed for the two honeys in which honeydew elements were found (numbers 28 and 29): 1328 mg kg^−1^ and 1023 mg kg^−1^, respectively. Although the heather honey contained less proline than the ones containing honeydew elements (956 mg kg^−1^), its content was still higher than in any of the other analyzed honeys. Pollen is considered the main source of amino acid; however, bees also contribute to the free amino acid content, which results in high variability of these components in honey, even from the same botanical origin [41]. Another amino acid that was found in honeys in noticeable amounts, but with great variation, was phenylalanine (Phe): in the range of 13 mg kg^−1^ to 294 mg kg^−1^, with the higher levels resulting in the existence of honeydew elements in honey. Higher glutamine (Gln), lysine (Lys), and glutamic acid (Glu) levels were observed in honeys containing major Cruciferae pollen amounts. These three amino acids, as well as arginine (Arg) and histidine (His), are known to be characteristic of rapeseed honeys [42]. 

### 3.4. Bioactive Compounds

#### 3.4.1. Polyphenols and Flavonoids

Polyphenols come to honey through plant nectar, propolis, and pollen [43]. The average total polyphenol value of the analyzed honeys was 41.9 mg GAE per 100 g, and the average total flavonoid content was 3.5 mg QE per 100 g (Table 2). The total polyphenol variability was much higher than total flavonoid variability. The total polyphenol content of polyfloral honeys was generally much lower than that of heather honey. The total polyphenol and flavonoid content in heather honey was almost twice as high as the average: 88.7 mg GAE per 100 g and 6.4 mg QE per 100 g, respectively. The average total polyphenol and flavonoid levels in honeys that contained honeydew elements were 62.5 mg GAE per 100 g and 5.1 mg QE per 100 g, respectively. The polyphenol content of the aforementioned honeys has also been found to be higher by other researchers [8,26].

A high correlation between total polyphenol and total flavonoid content was found (Table 3), which is consistent with the results of Escuredo et al. [43], A-Rahaman et al. [44] and Khalil et al. [45]. 

The electrical conductivity and free acidity levels seemed to have stronger connections to polyphenols by calculated correlations than any other physicochemical honey property. Moreover, a high correlation was found between polyphenol content and honey color. The polyphenol concentration increased with decreasing honey lightness (L*) and with increasing honey redness (a*). This is in agreement with Kuś et al. [11] and Bertoncelj et al. [3]. Of all the amino acids, mostly alanine (Ala) and proline (Pro), and to a lesser extent glutamic acid (Glu), glycine (Gly), threonine (Thr), and valine (Val), most affected the antioxidant honey properties (Table 5). The highest correlation was found between alanine and total polyphenol content. The identified polyphenols showed the highest intensities of caffeic acid, coumaric acid, and abscisic acid, and its derivatives. Higher intensities were also detected in shikimic acid, 4-hydroxybenzoic acid, salicylic acid, quercetin, kaempferol, ferulic acid, and its derivatives.

**Figure 1:**
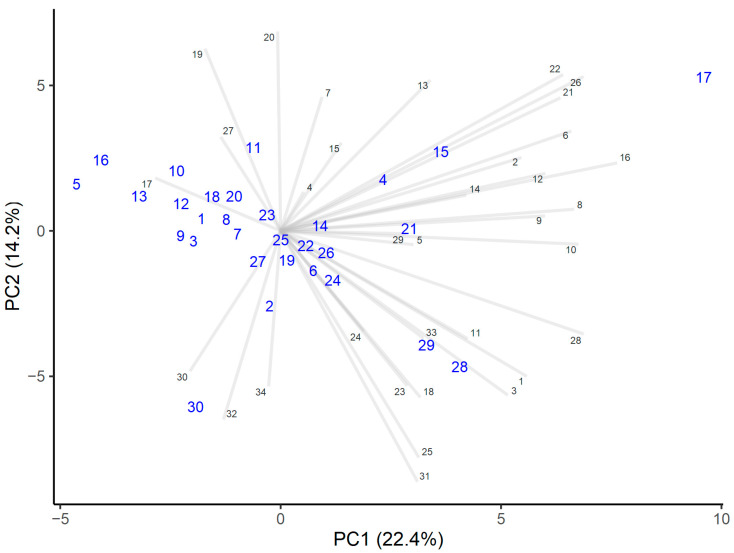
PCA analysis of the intensities of polyphenols. Honey samples are marked by larger blue numbers and polyphenols by smaller numbers marked black.

Protocatechuic acid can be considered a marker for honeydew honey, and distinguishes honeydew honey from polyfloral honeys [46]. This study showed that even if a honey contained only traces of honeydew elements, the protocatechuic acid intensities were significantly higher than in the other honeys analyzed (Figure 1).

Heather honey differed in terms of higher levels of cinnamic acid, myricetin, and abscisic acid derivatives D_2_ and D_3_. The levels of these components were also higher in those honeys that contained only minor heather pollen levels (honeys numbers 4 and 15, with 3% and 2% pollen, respectively). Therefore, higher cinnamic acid, myricetin, and abscisic acid derivative levels seemed to be characteristic of the heather honeys, and that is in accordance with other research [9,39].

Honey number 30 had higher intensities of flavonoids, such as galangin and chrysin, the latter indicating the presence of propolis in honey [47].

Small apigenin levels have been found only in rapeseed honeys and polyfloral honeys [5], and quercetin and kaempferol only in *Brassica* honeys [47], as botanical origin markers. Apigenin was present in all of the analyzed honeys, although in small amounts, and quercetin and kaempferol were found in similar amounts, which was because all honeys consisted of *Brassica* pollen to some extent. 

#### 3.4.2. Antioxidant Activity

The levels of water-soluble antioxidants (ACW), such as flavonoids, ascorbic acid and amino acids, and lipid-soluble antioxidants (ACL), such as tocopherol, carotenoids, and tocotrienols [29], were determined. The average water-soluble antioxidant level was 115.2 mg AAE per 100 g, and the average lipid-soluble antioxidant level was 24.2 mg TE per 100 g (Table 2). The analysis showed that the water-soluble antioxidant composition was dominant over the lipid-soluble antioxidant composition. The ACW of different honeys varied greatly and the highest levels were in the two honeys that contained honeydew elements or heather honey—299.3 mg AAE per 100 g, 311.2 mg AAE per 100 g, and 245.3 mg AAE per 100 g—indicating higher antioxidant properties. However, the highest ACL content (60.7 mg TE per 100 g), which was twice as high as in other honeys, was again associated with heather honey. The ACW and ACL values correlated with the total polyphenol content of honey (Table 3), which indicated that phenolic compounds might be the principal components that affect honey antioxidant properties. This correlation has also been found by other researchers [3,11,29]. In addition, a high correlation has been found between antioxidant activity and honey color. The lightness of honey correlated well with ACL value, while the redness correlated better with ACW value and amino acid content.

### 3.5. Sensory Evaluation

**Figure 2:**
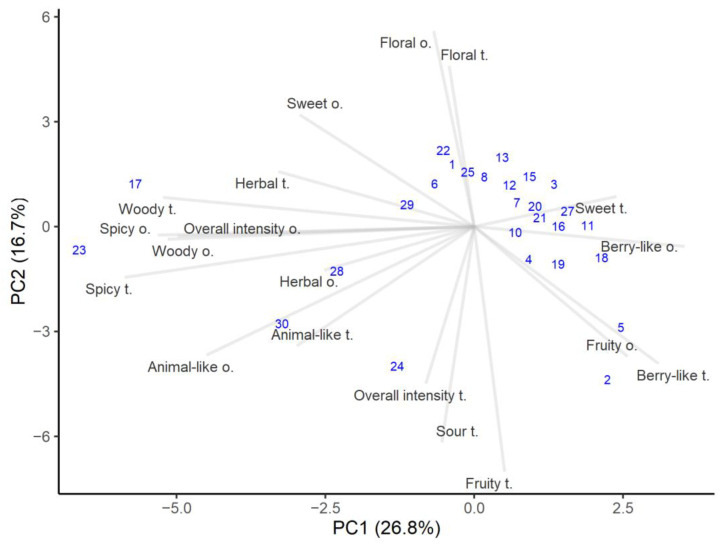
PCA analysis of the sensory attributes of honey samples. The number in parenthesis shows variance explained by the principal component.

Aroma and taste are important honey characteristics and depend on specific complex substances derived from its plant sources [40]. The sensory analysis results are presented in Figure 2. The first axis accounts for 26.8% of the variance and is positively related to berry-like and fruity but negatively related to spicy, woody, herbal, and animal-like. The overall odor intensity was influenced by woody and animal-like characteristics, which showed slightly higher correlations (*r* = 0.63 and *r* = 0.54, respectively). Meanwhile, the second axis explains the variance of 16.7% and is loaded positively for floral and sweet, and thus negatively for fruity, sour, and overall intensity in flavor. Overall taste intensity had the highest correlation with sour taste (*r* = 0.61). 

The sensory evaluation indicated that most of the honeys were grouped in the right top corner, showing higher floral, sweet, and berry-like characteristics. Honey sweetness can vary because sugars in honey have different sweetness levels [40]. These are typical quality honeys attributes, and floral and fruity notes are valued as more pleasant notes [48]. However, the overall odor and taste intensity tended to be lower for fruity samples compared to the samples with herbal woody and spicy notes. Most of the honey samples lacked of spicy, woody, and animal-like characteristics. Even if different honeys have the same floral source and the same number of pollen grains, the sensory characteristics of those honeys can be quite different. This could mean that honey properties and composition not only depend on the plant species that provide the nectar, but also on other factors, such as different locations, storage conditions, and even harvesting technology and conditions [49,50].

It is said that flavor is closely related to aroma [51] and this was corroborated by the calculated correlations, which for all flavor and odor attributes were above 0.5, except for sweetness. This means that when a certain odor characteristic was detected, with high probability it was recognized during tasting and vice versa. The highest correlation was found between spicy flavor and aroma notes (*r* = 0.93). It was interesting that the calculated correlation between overall aroma and flavor intensities was very low (*r* = 0.15), which means that these two attributes were independent of each other, depending on the honey composition.

Although most of the honeys had quite similar sensory profiles, some stood out for their distinct aroma and taste. Heather honey (number 17) and honey containing the highest amount of alder buckthorn pollen (number 23) had the highest spicy, woody, herbal, and animal-like notes. At the same time, the overall intensity was higher for both odor and flavor. Those honeys tasted less sweet than the others, and had the least berry-like aroma. Honeys containing traces of honeydew elements (numbers 28 and 29) showed high scores in animal-like and woody attributes, which is exactly characteristic of honeydew honeys [36].

The color of honey is related to its taste and darker honeys usually have stronger flavors [51]. In the present study, it was found that woody and sour attributes were most associated with lightness/darkness and redness/greenness. Thus, honeys with dark and reddish colors had stronger woody and sour flavors (the correlations in both cases were above *r* = 0.55).

## 4. Conclusions

The analysis of Estonian honeys indicated that the botanical origins are diverse and those honeys are polyfloral, with the most dominant pollen types being Cruciferae and Rosaceae, Salix, Trifolium, and Frangula alnus. The physicochemical values met all of the quality norms set by Directive 2001/110/EC, with some exceptions in moisture content. Among all of the analyzed honeys, heather honey and two polyfloral honeys stood out for their color, amino acid content, bioactive compounds, and organoleptic parameters. Although those polyfloral honeys contained only traces of honeydew elements, those traces still had significant influence on the honey properties. 

Those different honeys provide a good basis for comparing and evaluating typical polyfloral Estonian honeys. The total polyphenol and flavonoid contents, as well as antioxidant activity, varied greatly among the honey samples analyzed. These properties are strongly connected with honey color. Most analyzed honeys tended to be lighter in color, had rather mild flavor and aroma characteristics, had higher floral, sweet, and berry-like notes, and minimal spicy, woody, and animal-like notes. The overall intensity was quite low. Those honeys had relatively low bioactive and antioxidant properties. On the other hand, such properties were observed as being much higher in heather honey and honeys containing honeydew element traces. Those honeys were darker, more reddish and yellowish, had higher flavor and odor intensities, and had higher spicy, woody, herbal, and animal-like notes.

Honey lightness was strongly correlated with polyphenols and lipid soluble antioxidants. Honey redness seemed to be connected with water-soluble antioxidants and amino acid content. The honeys with higher electrical conductivity and free acidity levels tended to be richer in polyphenol content. Moreover, darker honeys tended to have stronger flavors, such as woody, and sour attributes were most associated with lightness/darkness and redness/greenness. Woody and animal-like attributes had the strongest effect on overall odor intensity, while sour attributes most affected overall flavor intensity.

Although more samples are needed, the results from of only a few distinctive honeys provide a good basis for further research and give primary knowledge of marker compounds for identifying the honeys that can be found, for example, among amino acids and polyphenols. 

## Figures and Tables

**Table 1:** The detected polyphenols and their derivatives (D) mass-to-charge ratios (*m*/*z*).

Number	Polyphenol	(M-H)-	Number	Polyphenol	(M-H)-
1	Shikimic acid	173.05	18	Salicylic acid	137.02
2	Gallic acid	169.01	19	Abscisic acid	263.13
3	Protocatechuic acid	153.02	20	Abscisic acid D_1_	263.13
4	Protocatechuic and gentisic acid D_1_	153.02	21	Abscisic acid D_2_	263.13
5	Chlorogenic acid	353.09	22	Abscisic acid D_3_	263.13
6	Chlorogenic acid D_1_	353.09	23	Luteolin	285.05
7	Catechin	289.08	24	Luteolin and kaempferol D_1_	285.05
8	4-hydroxybenzoic acid	137.02	25	Quercetin	301.03
9	Gentisic acid	153.02	26	Cinnamic acid D_1_	147.05
10	Caffeic acid	179.03	27	Cinnamic acid D_2_	147.05
11	Caffeic acid D_1_	179.03	28	Apigenin	269.05
12	Coumaric acid	163.04	29	Naringenin	271.07
13	Coumaric acid D_1_	163.04	30	Naringenin D_1_	271.07
14	Ferulic acid	193.05	31	Kaempferol	285.04
15	Ferulic acid D_1_	193.05	32	Chrysin	253.05
16	Myricetin	317.03	33	Chrysin D_1_	253.05
17	Morin	301.05	34	Galangin	269.05

**Table 2:** Physicochemical parameters and antioxidant activity of honey samples. HMF—hydroxymethylfurfural, F/G—fructose/glucose ratio, TPC—total phenolic content, TFC—total flavonoids content, ACW—water-soluble antioxidants, ACL—lipid-soluble antioxidants, L*—lightness/darkness, a*—greenness/redness, b*—blueness/yellowness.

Sample	Electrical Conductivity (mS cm^−1^)	Moisture (%)	Invertase Activity (U kg^−1^)	Free Acidity (mmol kg^−1^)	Diastase (Schade Unit)	HMF (mg kg^−1^)	Fructose (g 100 g^−1^)	Glucose (g 100 g^−1^)	F/G	TPC (mg GAE 100 g^−1^)	TFC (mg QE 100 g^−1^)	ACW (mg AAE 100 g^−1^)	ACL (mg TE 100 g^−1^)	L*	a*	b*
1	0.5	18.2	188.0	17.0	32.2	3.7	38.0	35.7	1.1	37.5	3.0	84.5	23.9	85.4	−0.3	43.4
2	0.4	19.8	126.0	40.0	33.8	6.8	36.5	34.8	1.1	53.6	3.4	118.4	31.2	72.8	5.8	44.0
3	0.3	16.8	133.0	20.0	29.2	8.4	38.1	33.1	1.2	38.3	3.2	65.1	20.1	84.9	1.3	38.6
4	0.6	20.0	206.0	35.0	48.6	6.1	37.8	32.1	1.2	53.9	3.5	55.5	17.3	82.3	2.3	42.3
5	0.3	20.0	124.0	18.0	22.0	5.0	37.3	37.8	1.0	28.3	2.5	37.8	18.4	88.1	−1.7	26.7
6	0.4	15.6	132.0	21.0	26.0	7.2	39.2	34.0	1.2	41.1	3.1	56.0	20.1	84.3	0.3	39.2
7	0.4	19.1	167.0	26.0	34.4	8.2	38.5	35.4	1.1	50.4	4.3	200.4	40.7	78.4	6.2	48.9
8	0.2	18.0	199.0	25.0	40.8	6.9	39.8	36.2	1.1	28.8	1.9	81.4	16.5	88.4	−1.1	29.1
9	0.3	19.5	50.6	38.0	19.6	19.5	36.8	35.6	1.0	34.8	3.3	66.5	16.9	85.6	−0.7	31.6
10	0.2	20.3	118.0	16.0	21.6	6.0	38.7	36.4	1.1	27.9	3.1	77.0	17.2	88.6	−1.6	27.5
11	0.2	19.8	114.0	22.0	26.3	11.5	36.5	35.3	1.0	30.9	3.1	96.8	20.5	85.4	−0.1	33.5
12	0.3	18.3	93.5	20.0	22.5	8.9	39.2	36.9	1.1	35.2	2.8	84.8	18.2	86.6	−0.6	36.2
13	0.3	16.4	63.9	18.0	21.1	11.8	38.3	35.5	1.1	29.8	2.1	78.4	18.1	88.0	−1.4	30.9
14	0.5	20.4	168.0	25.0	24.6	5.0	38.4	33.0	1.2	49.0	3.9	140.6	31.5	83.5	1.6	51.8
15	0.4	19.1	153.0	27.0	30.9	6.5	37.8	32.9	1.1	48.4	3.2	135.3	27.0	83.8	2.7	43.5
16	0.2	17.4	82.8	12.0	17.7	9.1	38.8	35.4	1.1	26.2	2.4	69.2	19.4	90.4	−1.1	29.6
17	0.7	20.4	114.0	39.0	58.8	7.8	39.7	31.6	1.3	88.7	6.4	245.3	60.7	65.3	9.1	37.8
18	0.2	18.5	74.1	21.0	25.6	9.2	39.5	37.0	1.1	33.7	3.5	87.0	20.7	84.4	0.3	35.8
19	0.3	19.3	119.0	23.0	24.9	7.2	37.5	36.8	1.0	40.3	3.3	113.9	21.4	85.4	2.0	44.4
20	0.4	17.6	184.0	16.0	28.1	5.6	38.4	34.2	1.1	38.7	3.0	87.5	17.8	86.3	0.4	40.6
21	0.7	17.8	182.0	23.0	22.5	9.4	38.5	30.2	1.3	52.6	4.3	105.9	22.8	81.2	3.0	46.5
22	0.5	18.2	102.0	20.0	15.4	5.1	41.1	29.2	1.4	40.2	3.8	85.7	14.4	85.5	0.6	40.8
23	0.8	15.6	168.0	14.0	21.1	6.5	41.5	29.9	1.4	46.4	4.2	82.0	16.6	83.5	1.9	46.6
24	0.7	19.9	172.0	31.0	39.1	9.2	44.5	37.4	1.2	50.7	5.7	176.9	31.9	82.8	3.8	47.6
25	0.3	20.7	145.0	23.0	26.4	8.4	46.4	37.9	1.2	35.6	2.8	105.4	25.2	87.1	−0.2	36.9
26	0.3	20.9	124.0	17.0	16.0	5.9	44.9	39.7	1.1	30.8	2.4	138.3	27.7	86.2	0.6	34.9
27	0.3	20.0	189.0	21.0	25.4	7.8	45.9	36.9	1.2	34.7	2.7	96.6	22.8	86.7	0.1	36.1
28	0.5	18.3	50.4	43.0	35.5	5.9	37.6	32.6	1.2	68.6	5.3	299.3	32.5	76.8	12.5	60.3
29	0.5	18.9	231.0	35.0	36.2	10.0	37.4	32.6	1.1	56.5	4.9	311.2	37.0	76.7	12.3	58.7
30	0.2	19.2	228.0	23.0	37.1	3.5	40.7	38.8	1.0	26.8	2.6	73.7	17.1	88.3	−1.2	25.6
Average	0.4	18.8	140.0	24.3	28.8	7.7	39.4	34.8	1.1	41.9	3.5	115.2	24.2	83.7	1.9	39.6
SD	0.2	1.4	49.8	8.2	9.7	3.0	2.7	2.7	0.1	13.8	1.0	67.9	9.6	5.3	3.8	8.8
Min.	0.2	15.6	50.4	12.0	15.4	3.5	36.5	29.2	1.0	26.2	1.9	37.8	14.4	65.3	−1.7	25.6
Max.	0.8	20.9	231.0	43.0	58.8	19.5	46.4	39.7	1.4	88.7	6.4	311.2	60.7	90.4	12.5	60.3

**Table 3:** Calculated correlations. EC—electrical conductivity, M—moisture, IA—invertase activity, FA—free acidity, D—diastase, HMF—hydroxymethylfurfural, F/G—fructose/glucose ratio, TPC—total polyphenol content, TFC—total flavonoids content, ACW—water-soluble antioxidants, ACL—lipid-soluble antioxidants, AA—amino acids, L*—lightness/darkness, a*—greenness/redness, b*—blueness/yellowness.

	EC	M	IA	FA	D	HMF	F/G	TPC	TFC	ACW	ACL	AA	L*	a*
M	−0.08													
IA	0.28	0.09												
FA	0.34	0.36	−0.04											
D	0.41	0.23	0.40	0.64										
HMF	−0.18	−0.05	−0.43	0.25	−0.17									
F/G	0.68	−0.21	0.20	−0.01	0.03	−0.17								
TPC	0.77	0.12	0.07	0.72	0.66	−0.06	0.41							
TFC	0.78	0.13	0.04	0.60	0.51	0.03	0.43	0.88						
ACW	0.42	0.24	0.06	0.62	0.46	0.01	0.17	0.73	0.75					
ACL	0.46	0.37	0.06	0.56	0.61	−0.02	0.14	0.80	0.72	0.80				
AA	0.39	0.07	0.04	0.73	0.54	−0.04	0.12	0.74	0.71	0.86	0.62			
L*	−0.62	−0.18	−0.02	−0.74	−0.65	−0.01	−0.24	−0.93	−0.80	−0.71	−0.85	−0.66		
a*	0.55	0.11	0.08	0.73	0.54	−0.02	0.23	0.85	0.80	0.92	0.75	0.89	−0.85	
b*	0.65	−0.06	0.20	0.47	0.24	−0.12	0.36	0.69	0.69	0.71	0.46	0.71	−0.57	0.79

**Table 4:** The amino acid composition of honeys. Ala—alanine, Asp—aspartic acid, GABA—gamma aminobutyric acid, Gln—glutamine, Glu—glutamic acid, Gly—glycine, Ile—isoleucine, Leu—leucine, Lys—lysine, Phe—phenylalanine, Pro—proline, Ser—serine, Thr—threonine, Tyr—tyrosine, Val—valine.

Sample	Ala	Asp	GABA	Gln	Glu	Gly	Ile	Leu	Lys	Phe	Pro	Ser	Thr	Tyr	Val
1	9	7	4	18	19	2	4	6	13	27	334	8	5	8	8
2	11	11	5	26	20	3	7	13	22	73	552	11	5	9	9
3	12	15	6	48	27	3	9	13	19	98	512	15	8	9	13
4	10	14	2	23	18	3	3	4	20	17	622	12	7	7	8
5	4	6	2	28	9	0	2	1	10	26	257	5	2	8	4
6	9	7	4	30	13	2	6	8	18	283	426	10	5	33	8
7	11	10	1	42	23	2	18	28	21	92	643	11	6	22	12
8	8	6	5	24	15	2	4	6	20	19	543	9	5	11	7
9	10	13	6	53	22	3	8	8	20	36	399	10	5	8	6
10	7	10	4	31	15	2	4	5	19	15	290	9	5	4	7
11	8	8	5	29	10	3	4	4	21	16	389	10	5	6	6
12	8	9	5	35	15	3	6	11	18	83	367	12	6	9	8
13	8	11	4	44	20	2	7	8	15	20	350	12	7	7	10
14	11	9	4	35	18	3	11	27	15	230	447	10	8	18	12
15	12	10	7	42	20	4	25	43	20	27	480	12	10	38	15
16	6	7	4	24	12	2	4	0	15	19	307	9	5	6	7
17	24	19	14	28	32	7	9	11	31	33	956	20	14	13	16
18	8	8	6	35	15	4	5	9	23	36	430	11	5	8	7
19	12	11	6	44	24	4	7	8	36	49	638	13	7	10	10
20	9	13	2	39	15	2	4	5	15	22	492	11	5	5	8
21	13	19	3	13	22	2	3	5	11	22	661	12	5	4	7
22	10	12	3	9	16	3	3	5	8	18	471	11	4	6	6
23	10	9	3	3	21	2	2	5	2	16	375	8	3	8	4
24	18	46	4	41	49	7	9	10	26	31	757	31	13	13	15
25	8	13	3	33	14	2	4	5	21	30	475	11	4	6	7
26	9	15	6	37	18	1	4	3	23	13	525	12	5	4	8
27	7	7	4	24	12	2	4	4	14	36	320	8	4	9	6
28	26	26	9	56	37	7	16	32	33	294	1328	28	11	28	19
29	18	20	6	46	29	4	9	20	30	205	1023	18	7	18	13
30	9	16	5	36	22	3	15	22	33	42	589	15	6	19	11

**Table 5:** Calculated correlations between amino acids and TPC, TFC, ACW, ACL. TPC—total polyphenol content, TFC—total flavonoids content, ACW—water-soluble antioxidants, ACL—lipid-soluble antioxidants.

Amino Acids	Ala	Asp	GABA	Gln	Glu	Gly	Ile	Leu	Lys	Phe	Pro	Ser	Thr	Tyr	Val
TPC	0.88	0.47	0.54	0.07	0.63	0.73	0.33	0.41	0.33	0.37	0.77	0.58	0.71	0.37	0.67
TFC	0.86	0.67	0.42	0.09	0.77	0.78	0.27	0.33	0.32	0.34	0.74	0.71	0.69	0.27	0.63
ACW	0.85	0.54	0.51	0.40	0.66	0.67	0.47	0.55	0.56	0.49	0.87	0.70	0.63	0.42	0.74
ACL	0.72	0.38	0.59	0.22	0.54	0.61	0.39	0.42	0.46	0.28	0.63	0.50	0.68	0.33	0.66

## Data Availability

The data presented in this study are available on request from the corresponding author.

## Supplementary Materials

The following are available online at https://www.mdpi.com/2304-8158/10/3/511/s1, Figure S1: UV chromatograms of amino acids of standard (black line) and honey sample number 1 (blue line).
