# Supplementary material for: The Composition, Physicochemical Properties, Antioxidant Activity, and Sensory Properties of Estonian Honeys
Source: Foods. 2021 Mar 1;10(3):511. doi: 10.3390/foods10030511 (PMC8002063; doi:10.3390/foods10030511)
Supplement: Supplementary file 1 [file foods-10-00511-s001.pdf]

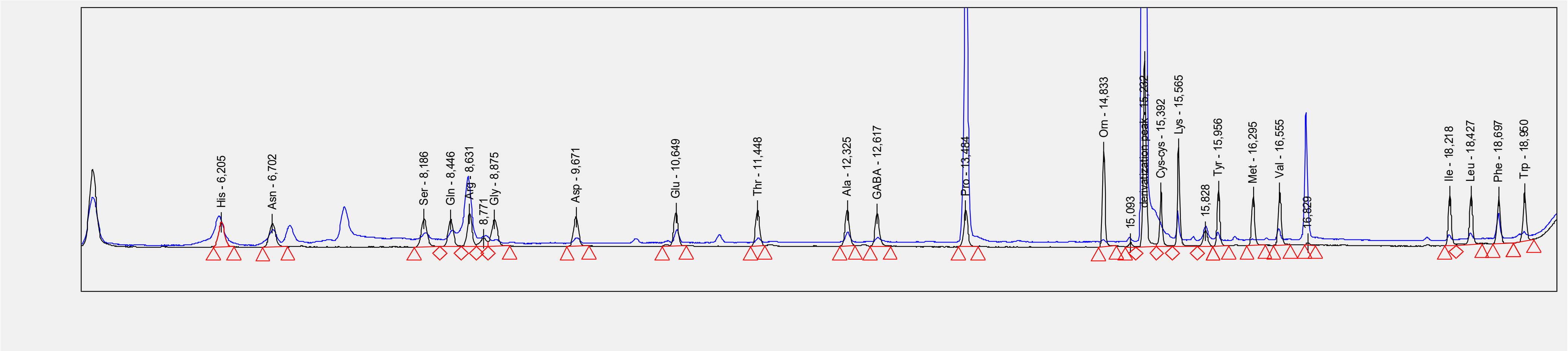

Supplementary Figure S1. UV chromatograms of amino acids of standard (black line) and honey sample number 1 (blue line)
